# Getting the Akt Together: Guiding Intracellular Akt Activity by PI3K

**DOI:** 10.3390/biom9020067

**Published:** 2019-02-16

**Authors:** Ivan Yudushkin

**Affiliations:** Department of Structural and Computational Biology, University of Vienna, Max F. Perutz Laboratories Vienna BioCenter, Campus Vienna Biocenter 5, Rm. 1.624, 1030 Vienna, Austria; ivan.yudushkin@univie.ac.at; Tel.: +49-1-4277-74340

**Keywords:** PI3K, protein kinase Akt, cellular signaling

## Abstract

Intracellular signaling pathways mediate the rapid response of cells to environmental cues. To control the fidelity of these responses, cells coordinate the activities of signaling enzymes with the strength, timing, and localization of the upstream stimuli. Protein kinase Akt links the PI3K-coupled receptors to cellular anabolic processes by phosphorylating multiple substrates. How the cells ensure that Akt activity remains proportional to upstream signals and control its substrate specificity is unclear. In this review, I examine how cell-autonomous and intrinsic allosteric mechanisms cooperate to ensure localized, context-specific signaling in the PI3K/Akt axis.

## 1. Introduction

Protein kinase B/Akt belongs to the AGC family of serine/threonine kinases, sharing common structural organization and regulatory mechanisms [1]. In mammalian cells, Akt is expressed as three isoforms, Akt1, -2, and -3 (also referred to as PKBα, −β, and −γ), which display high sequence identity and overlapping substrate specificity [2]. Functionally, Akt links the cell surface receptors coupled to phosphatidylinositol-3 kinase (PI3K) to the signaling pathways controlling cellular anabolic processes [3] by phosphorylating ~300 various cellular substrates [4,5]. Akt therefore plays an essential role in promoting glucose uptake and cell growth, survival, and proliferation in response to cytokines and growth factors.

The main function of Akt—coordination between the cellular pathways regulating energy-costly anabolic processes with the extracellular growth and survival cues—requires that its activity remains proportional to the activating stimuli. In the case of Akt, these upstream signals converge on PI3K. Uncoupling of extracellular cues from PI3K activation often leads to accumulation of the PI3K product, the membrane lipid phosphatidylinositol-3,4,5-trisphosphate (PI(3,4,5)P_3_) and results in Akt hyperactivation and uncontrolled cell growth. Such dysregulation of the PI3K/Akt pathway is observed in many human cancers (reviewed in [6]), indicating that in normal cells Akt activity is tightly controlled.

In cells, the kinase activity of Akt is controlled by both intramolecular (“intrinsic”) and cell-autonomous mechanisms. We will first consider how posttranslational modifications and lipids control Akt activity, overview the several models of Akt regulation inside cells, and finally discuss the physiological relevance of tight coupling between PI3K and Akt.

## 2. Akt-Intrinsic Regulatory Mechanisms

### 2.1. Posttranslational Modifications

Of the many posttranslational modifications reported for Akt (reviewed in [7]) essential for its kinase activity is phosphorylation of the two critical residues: T308 in the activation loop of the kinase and S473 in the hydrophobic C-terminal extension. Phosphorylation of T308 by PDK1 positions the residues involved in the catalysis [8,9] and is absolutely required for the activity. Phosphorylation of S473 in the C-terminus by mTOR complex 2 (mTORC2) further increases the kinase activity four- to 10-fold in cells [10,11] and up to 18-fold (with a staggering 450-fold increase in catalytic efficiency) in vitro [12]. Unlike other AGC kinases, such as PKC or SGK, which require phosphorylation of their hydrophobic motifs to promote activation by PDK1, phosphorylation of S473 is not essential for the Akt kinase activity [12,13,14]. Yet, docking of S473 on the N-lobe of the Akt kinase domain stabilized the critical αC helix, thereby increasing the specific activity of the isolated kinase domain [8] and enhancing its interaction with PDK1 [13]. Indeed, it appears that phosphorylated S473 (as well as S477 and T479) enhance the rate of PDK1-mediated phosphorylation of T308 [12], suggesting a priming role for S473 in Akt activation. This so-called PIF pocket (PDK1-interacting fragment) mechanism, which typically recruits other AGC kinases to PDK1 [15], was proposed to supply an alternative route for Akt activation in the absence of PI3K signals, such as upon DNA damage in the nucleus [16]. However, in which order Akt regulatory sites are phosphorylated and to what extent the PIF pocket mechanism contributes to Akt activation in cells is currently unclear.

Two more sites in the Akt C-terminus have gained attention in the recent years. Phosphorylation of S477 and T479, most likely by the Cdk2/cyclin A complex, further increased Akt catalytic efficiency by two-fold [12,17]. Phosphorylated S477/T479 have been proposed to bind the Akt activation loop, thereby displacing the PH domain and relieving the autoinhibitory conformation. While biochemical evidence suggests the involvement of these residues in Akt regulation [12], further studies would be required to test the relevance of their phosphorylation in the cellular context.

Several other posttranslational modifications have been proposed to regulate Akt activity. Several serine/threonine (e.g., Tbk1, Ikkε) and tyrosine kinases (such as Ack1, Src and Ptk6) were reported as alternative Akt activators (reviewed in [18]). Hydroxylation of prolines 135 and 313 by the oxygen-dependent hydrolylase EglN1 [19] promoted interaction with von Hippel-Lindau protein (pVHL) and inhibited Akt. A recent study demonstrated that in a number of melanoma cell lines, dual inhibition of mTORC1/2 results in mTORC2-independent and PDK1- and PI3K-dependent Akt reactivation via an as yet unidentified mechanism dependent on IGF1R and integrin signaling [20]. To what extent these modifications are relevant for Akt activity in cells and tissues remains to be established.

### 2.2. Akt Allosteric Regulation by Lipid Binding

Binding to PI(3,4,5)P_3_ and PI(3,4)P_2_ via the N-terminal pleckstrin homology (PH) domain is critically required for Akt kinase activity. First, accumulation of PI(3,4,5)P_3_ at the plasma membrane following growth factor-induced PI3K activation recruits Akt to its activating kinases, PDK1, and mTORC2. PDK1, which also has a PH domain, is both recruited to and directly activated by PI(3,4,5)P_3_ binding at the plasma membrane, resulting in phosphorylation of Akt at T308 [21,22]. Unlike PDK1, mTORC2 activity at the plasma membrane is constitutive and PI3K-insensitive [23], so that membrane recruitment is sufficient for Akt phosphorylation on the mTORC2 site, S473. In line with the role of PI(3,4,5)P_3_ in Akt recruitment to its activating kinases at the membrane, PI3K inhibition or mutations and posttranslational modifications in the PH domain interfering with its membrane binding all potently reduce phosphorylation of Akt and its substrates [24,25,26,27]. Conversely, Akt mutants displaying increased membrane association (myristoylated v-Akt, E17K, E40K, etc.) are both hyperphosphorylated and oncogenic [28,29], indicating that membrane binding is both necessary and sufficient for Akt activation.

Besides its role in recruiting Akt to its activating kinases at the cellular membrane, PI(3,4,5)P_3_ binding to the PH domain also allosterically regulates Akt kinase activity. Thus, deletion of the PH domain was shown to increase Akt phosphorylation on T308 by PDK1 and enhance the basal kinase activity both in cells and in vitro [16,21]. Furthermore, it was recently shown that binding of PI(3,4,5)P_3_ and PI(3,4)P_2_ to the PH domain was required to stimulate kinase activity of phosphorylated Akt [14]. In agreement with previous reports [16,21], truncation of the PH domain or its substitution for the PH domain of a non-related tyrosine kinase Btk resulted in constitutive, PI(3,4,5)P_3_-independent kinase activity, quantitatively identical to the kinase activity of the wild type Akt in presence of PI(3,4,5)P_3_- or PI(3,4)P_2_-containing liposomes. This provided the first biochemical demonstration of the existence of the PH domain-dependent allosteric switch in Akt.

**Figure 1:**
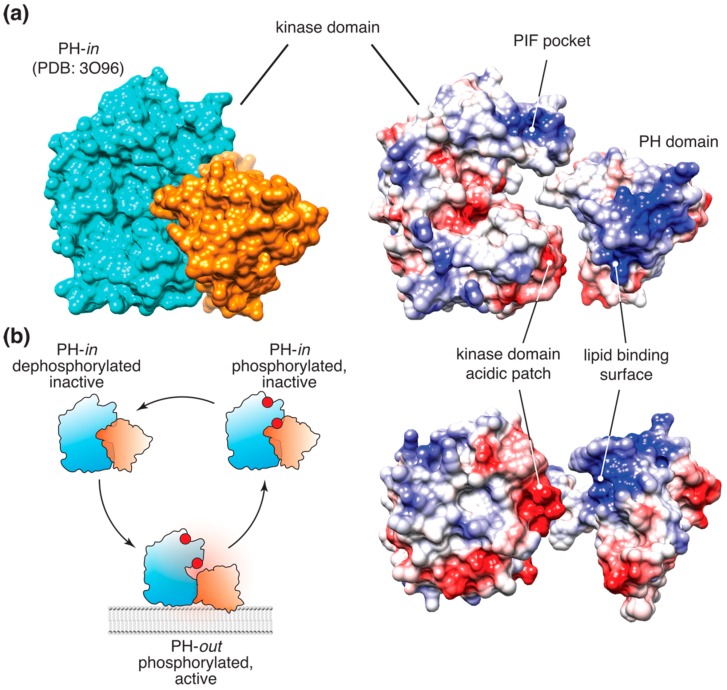
Akt allosteric mechanism. (**a**) (top left) Surface representation of the autoinhibited, “PH-in” conformation of Akt [33]. (right) Surface electrostatic representation of Akt. The PH and kinase domains are separated to highlight the allosteric interface formed by the complementary acidic patch on the C-lobe of the kinase and the PI(3,4,5)P_3_-binding basic patch on the PH domain; (**b**) The allosteric switch model of Akt activation in cells. Only membrane-bound Akt in the extended “PH-out” conformation is catalytically active. Dissociation from PI(3,4,5)P_3_- or PI(3,4)P_2_-containing membranes results in re-formation of the autoinhibited “PH-in” conformation and rapid dephosphorylation of Akt.

This allosteric mechanism, originally proposed by Stokoe et al. [21] and later developed using a conformational FRET reporter by Calleja et al. [30,31,32], was further substantiated by the crystal structure of the full-length Akt in complex with the allosteric inhibitor VIII [33]. In this structure, the inhibitor promoted tight packing of the PH domain against the kinase (Figure 1a), resembling the hypothetical “PH-in” conformation, proposed by Calleja et al. as the opposite of the extended “PH-out” conformation. The latter was originally defined as the conformation corresponding to the low-lifetime population of the Akt FRET reporter, accumulating at the membrane and in the cell interior following growth factor stimulation [30]. The crystal structure of the putative “PH-in” conformation prompted an extensive investigation of the proposed allosteric interface [34]. Of the mutations examined, only three were shown to be sufficient to induce cell transformation. An E17K mutation in the PH domain was previously shown to result in stronger membrane association of Akt and sufficient to induce cellular transformation [29,35]. The other transforming mutation at the proposed allosteric interface, D323A, is located in an acidic patch on the C-lobe of the kinase, near the Akt active site. Notably, in the Akt crystal structure stabilized by inhibitor VIII, D323 forms an ionic bridge with lysine K14, which is absolutely required for PI(3,4,5)P_3_ binding (Figure 1a). In fact, in the full-length Akt crystal structure stabilized by the inhibitor VIII [33], the PI(3,4,5)P_3_ binding surface of the PH domain is almost entirely buried through extensive (>1500 Å^2^) interactions with the kinase domain, suggesting that the PI(3,4,5)P_3_-bound and the autoinhibited apo-conformations of Akt are mutually exclusive.

Careful biochemical examination of Akt regulation allowed us to propose a model (Figure 1b) where binding of PI(3,4,5)P_3_ or PI(3,4)P_2_ to the PH domain of Akt relieves the autoinhibitory allosteric communication between the PH and kinase domain, inducing a nearly 40-fold increase in the affinity of the kinase to peptide substrates [14]. Notably, this allosteric mechanism appears to operate even in the presence of T308 and S473 phosphorylation, indicating that the latter is necessary, but not sufficient for full Akt activity. This conclusion, however, was recently challenged by a detailed biochemical study, which reported no effect of lipids on the activity or substrate binding of stoichiometrically phosphorylated Akt in vitro [12]. A possible explanation for these conflicting data could be that Akt activity assays used by Chu et al. [12] were done in the presence of saturating Mg^2+^ concentration, which, as we found, chelates PI(3,4,5)P_3_ and could mask the allosteric effect of the lipids on the kinase [14]. An exciting alternative would be that lipid binding differentially affects Akt phosphorylation by PDK1 and mTORC2. A plausible scenario would be S473 phosphorylation priming Akt activation and/or helping it bypass the requirement for PI(3,4,5)P_3_ binding, as was proposed earlier [16]. Currently, there is no strong biochemical evidence to support or refute this hypothetical mechanism.

### 2.3. Other Possible Mechanisms

Several alternative mechanisms could also contribute to the Akt allosteric activation. Thus, Akt was reported to be a weak client of the molecular chaperones Hsp90 and/or Cdc37 [36,37,38]. A few early publications (reviewed in [39]) reported binding of the protooncogene product Tcl1 to the Akt PH domain, which resulted in increased phosphorylation of Akt substrates Bad and Gsk3β in vitro and accumulation of Akt in the nuclei. Interestingly, Tcl1 binding was mapped to the surface of the Akt PH domain opposite to the PI(3,4,5)P_3_ binding site [40]. It is tempting to speculate that such binding could potentially overcome the PH domain-dependent allosteric mechanism, thereby surpassing the requirement for PI(3,4,5)P_3_ binding. An unexpected finding that a long intergenic non-coding RNA, *LINK-A*, could bind to the Akt PH domain and to PI(3,4,5)P_3_ [41] indicates the existence of further alternative mechanisms of Akt activation in cells. An exact biochemical characterization would be required to test whether and how binding of Hsp90/Cdc37, Tcl1, or *LINK-A* activates Akt in the cellular context.

## 3. Cell-Autonomous Akt Control Mechanisms

The most relevant experiments for Akt’s regulation and its role in cellular physiology are those conducted inside cells. However, even after three decades of active research, many questions remain unanswered.

### 3.1. Classical, or Diffusive, Model

In quiescent cells, both endogenous and GFP-tagged Akt localize to the nucleus and the cytosol, as can be seen using conventional imaging. Treatment with cytokines or growth factors coupled to PI3K activation results in transient accumulation of Akt at the plasma membrane and intracellular vesicles [14,30,42,43]. This accumulation peaks between 2 and 5 min after stimulation. After ~15 min, very little, if any, Akt can be detected at the plasma membrane.

**Figure 2:**
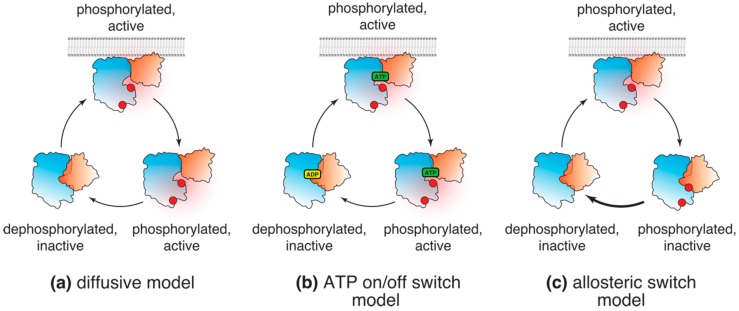
Models of Akt (in)activation in cells. (**a**) According to the diffusive model, following phosphorylation, Akt dissociates from the plasma membrane and diffuses throughout the cell in the active conformation, phosphorylating its substrates; (**b**) “ATP on/off” model suggests that Akt freely diffuses in its active, phosphorylated conformation as long as it is ATP bound. Substrate phosphorylation and conversion of ATP to ADP results in Akt dephosphorylation and inactivation; (**c**) The allosteric lipid switch model proposes that the active, “PH-out” conformation of Akt is limited to cellular membranes displaying PI(3,4,5)P_3_ (or PI(3,4)P_2_). Membrane dissociation leads to autoinhibitory conformation and rapid dephosphorylation of Akt by cellular phosphatases.

Unlike transient membrane accumulation, phosphorylation of Akt and its substrates in response to growth factors, such as insulin or IGF, is typically sustained up to 1–2 hours after stimulation [14,44,45,46]. Combined, these two observations gave rise to a classical model of Akt activation. According to this model, following its transient accumulation and phosphorylation at the plasma membrane, Akt dissociates from the membrane and freely diffuses throughout the cell interior in its active, phosphorylated form (Figure 2a).

Several lines of evidence are usually presented to support this model. It was postulated that phosphorylated Akt corresponds to the active, “PH-out” conformation, which could then be monitored using a conformational FRET probe [30]. Indeed, despite a very low (<9%) FRET efficiency, treatment with PDGF triggered slow accumulation of the “PH-out” conformation of the Akt FRET reporter at the plasma membrane and in the cell interior [30]. These data nicely complemented an earlier study on phosphorylation dynamics of another FRET probe by the endogenous Akt [47]. Both studies demonstrated gradual accumulation of active Akt in the cell interior and in the nucleus, which is consistent with the classical model and have been extensively cited in its support. The crystal structure of the full-length Akt in complex with the inhibitor VIII [33], reminiscent of the postulated “PH-in” conformation, provided further credence to the results of the FRET study. Furthermore, follow-up studies using ATP analog inhibitors have proposed that the active form of Akt is stabilized by interactions of phosphorylated T308 with residues in the Akt active site and the hydrophobic C-terminal extension [48,49,50]. These observations gave rise to the “phosphatase shielding cage” model [48], where a network of interactions protected phosphorylated Akt from cytosolic phosphatases.

### 3.2. “ATP On/Off Switch” Model

An extension of the diffusive model was an elegant hypothesis proposed by Lin et al. [49]. It is based on the fact that, while ATP-competitive inhibitors induce paradoxical hyperphosphorylation of Akt in cells [48,51,52], ADP analogs fail to do so [49]. In line with the “phosphatase shielding cage” model, Lin et al. [49] concluded that ATP and ATP analogs protect T308 and S473 from dephosphorylation both in vitro and in cells. They proposed that exchange of ATP for ADP in the active site upon catalysis promotes Akt inactivation by interfering with the “phosphatase shielding cage” mechanism, making ADP-bound Akt a better substrate for cellular phosphatases (Figure 2B).

This elegant and appealing model suggesting that Akt is likely inactivated following a single round of substrate phosphorylation was recently challenged. We showed that both wild-type and kinase-inactive Akt mutant displayed similar rates of dephosphorylation upon PI3K inhibition. This simple result demonstrated that exchange of ATP for ADP in Akt catalytic cycle has no effect on the rate of Akt dephosphorylation. Rather, membrane dissociation appears to be the dominant mechanism triggering Akt inactivation in cells [14]. It is, however, possible that ATP binding could stabilize phosphorylated Akt when it is membrane-bound. Notably, in previous studies, ATP analogs only induced hyperphosphorylation of membrane-bound Akt, either myristoylated or in the presence of growth factors [48,49,52], suggesting that ATP-competitive inhibitors trap Akt in its active, PI(3,4,5)P_3_-bound conformation. Supporting this hypothesis, an ATP analog induces membrane accumulation of the endogenous Akt [52], supporting the argument that active Akt is indeed membrane-bound.

### 3.3. Allosteric Switch Model

While this classical diffusive model explained the existing empirical data well, it also posed several questions. First, the presence of an active kinase directly implicated in cell survival and proliferation inside cells for hours after the initial growth factor pulse raises concerns regarding how its activity is kept under control. Second, Akt phosphoproteomic analysis [5] demonstrated that its substrates display distinct phosphorylation kinetics, incompatible with distributive phosphorylation implied by the classical model. Finally, simple diffusion at 12 μm^2^/sec [14] would allow Akt to explore distances within 20 μm (the diameter of a typical HeLa cell) in well under 5 seconds, whereas FRET reporters [30,47] indicated the half-time of Akt activation on the order of 3–5 minutes, suggesting that diffusion of active Akt in cells is restrained.

Central to the classical model is the question of whether phosphorylated Akt is sufficiently long-lived to diffuse through the cytosol in its active conformation. Akt activity inside cells is limited by phosphatases, such as PP2A and PHLPP1/2 [53,54,55], which inactivate Akt by removing the phosphate groups from T308 and S473. If the classical model holds true, then phosphorylated Akt will persist even upon depletion of its lipid ligand PI(3,4,5)P_3_. However, the rates of Akt dephosphorylation and PI(3,4,5)P_3_ depletion upon acute PI3K inhibition were indistinguishable, suggesting that Akt inactivation is rate-limited by PI(3,4,5)P_3_ lifetime [14]. Furthermore, using a PI3K-orthogonal reversible chemical heterodimerization approach, we demonstrated that Akt dephosphorylation follows the kinetics of its dissociation from the membrane, indicating that cellular phosphatases rapidly inactivate Akt in the cytosol [14]. These observations are reminiscent of the “dephosphorylation by default” model proposed for Akt and other signaling kinases [56,57], and demonstrate that very little, if any, cytosolic Akt is phosphorylated.

These results not only challenged the classical model, but also demonstrated that phosphorylated, active Akt is partitioned to cellular membranes containing PI3K products (Figure 2c). Three lines of evidence support that conclusion. First, dissociation from PI(3,4,5)P_3_ and PI(3,4)P_2_ at the cellular membranes led to rapid Akt dephosphorylation, while interference with the Akt allosteric switch significantly slowed down its dephosphorylation in cells [14]. These results indicate that dissociation from the membranes and formation of the autoinhibitory interface between the PH and kinase domains exposes phosphorylated T308 and S473 to cellular phosphatases. Second, our biochemical data imply that upon dissociation from the membrane and re-establishment of the autoinhibitory “PH-in” conformation, Akt affinity for its peptide substrates decreases at least 8-fold [14]. Finally, using a short peptide derived from a classical Akt substrate FoxO3a, which was shown to specifically interact with the phosphorylated, ATP-loaded Akt [48,58], we have shown that active Akt displays diffusive behavior of a membrane-bound protein. Taken together, these observations demonstrate that the kinase-intrinsic allosteric and cell-autonomous mechanisms cooperate to couple Akt to PI3K activity and restrict it to cellular PI(3,4,5)P_3_- and PI(3,4)P_2_-containing membranes.

## 4. Physiological Relevance of PI3K/Akt Coupling

Partitioning of active Akt to PI(3,4,5)P_3_- or PI(3,4)P_2_-containing membranes may have important implications for regulation and fidelity of Akt-controlled signaling processes in cells. First and foremost, coupling of Akt activity to lipid binding ensures that its activity remains proportional to the upstream signals, which converge on PI3K. Conversely, uncoupling between lipid binding and Akt activity could induce cellular transformation. Indeed, an activating E17K mutation resulting in increased membrane association of Akt was identified in human colorectal, ovarian, and breast cancers [29] and in patients with megalencephaly and Proteus syndrome [59,60]. Similarly, myristoylated Akt of murine leukemia virus is constitutively hyperactive and induces leukemia and cancer in peripheral organs [61]. A D323H mutation at the allosteric interface results in cellular transformation and was identified in urinary carcinoma [34,62]. In all these cases, pathological Akt hyperactivation is likely due to the uncoupling of Akt membrane binding and activity from PI3K, strongly suggesting that in untransformed cells the allosteric switch mechanism safeguards Akt against disproportionate activation.

Secondly, biochemical coupling with PI(3,4,5)P_3_ and PI(3,4)P_2_ allows cells to control the kinetics of Akt activation and quenching by modulating the rates of synthesis and degradation of PI(3,4,5)P_3_ and PI(3,4)P_2_ and their precursors. Thus, accumulation of PI(3,4,5)P_3_ at the plasma membrane following growth factor stimulation accounts for a transient Akt activity burst [63]. SHIP2-catalyzed dephosphorylation of PI(3,4,5)P_3_ at the plasma membrane and accumulation of the longer-lived PI(3,4)P_2_ at the plasma membrane and early endosomes [63] was associated with the long-term Akt activation. Similarly, the sustained level of PI(3,4)P_2_ in mouse embryonic fibroblasts lacking inositol-polyphosphate 4-phosphatase 1 resulted in prolonged phosphorylation of Akt in response to EGF, which typically induces only transient Akt activation [64]. Consistently, knockout of class II *PI3K-C2γ*, which is recruited to Rab5/Appl1-positive early endosomes in response to insulin, led to weaker and shorter insulin-induced Akt phosphorylation in liver and primary hepatocytes [65], providing further evidence that the sustained Akt activation on the plasma membrane and endosomes is defined primarily by PI(3,4)P_2_ dynamics.

Clathrin-mediated endocytosis of PI3K-coupled surface receptors also serves the dual function of sustaining Akt-activating phosphoinositides in the cell interior and ensuring the efficient signal termination. Thus, a small GTPase Rab5 was shown to be required for the recruitment of the PI3K regulatory subunit to insulin receptor substrate 1 and Akt phosphorylation on early endosomes [66], demonstrating that endosomes help sustain Akt activity. Endosomes were shown to display activities capable of producing PI(3,4)P_2_, either from phosphorylation of PI(4)P by class II PI3K [67,68,69] or upon dephosphorylation of PI(3,4,5)P_3_ by SHIP phosphatases [63]. Consistent with this notion are multiple reports linking endocytosis with phosphorylation of Akt and its substrates [70,71,72]. Conversely, dissociation of Appl1 and recruitment of the lipid 3-phosphatase PTEN to endosomes lead to Akt dephosphorylation [73,74,75], indicating that in addition to sustaining Akt phosphorylation, endosomal maturation and trafficking lead to eventual quenching of PI3K/Akt signaling.

**Figure 3:**
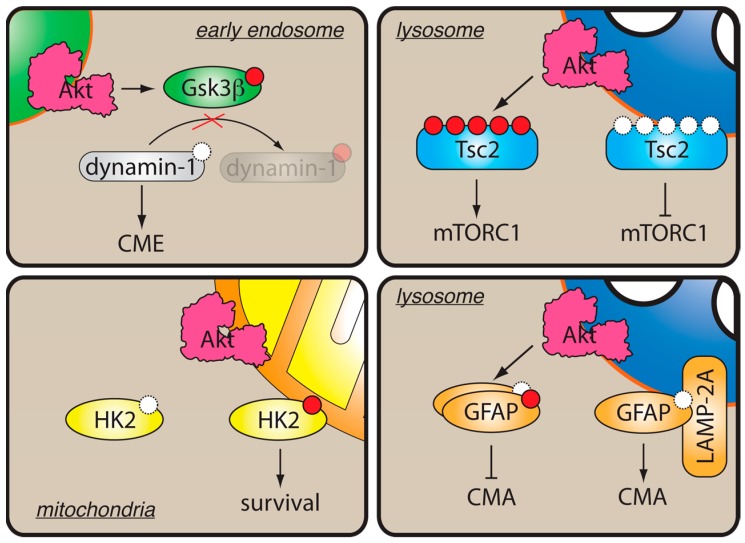
Localized Akt activity in cells. Membrane binding helps restrict Akt substrate specificity. On early endosomes, phosphorylation and inactivation of Gsk3β by Akt stimulates dynamin1-dependent clathrin-mediated endocytosis (CME). On lysosomes, phosphorylation of Tsc2 by Akt results in its dissociation and activation of mTORC1. Phosphorylation of GFAP by Akt on lysosomes promotes its dissociation from LAMP-2, thereby inhibiting chaperone-mediated autophagy (CMA). At the mitochondrial level, phosphorylation of hexokinase-II (HK2) by Akt induces HK2 binding to mitochondrial membrane and promotes cell survival.

Restriction of Akt activity to subcellular membrane compartments could help define the specificity of Akt signaling by targeting the kinase to a particular set of substrates (Figure 3). Thus, knockdown of the endosomal protein Appl1 in zebrafish resulted in decreased phosphorylation of the Akt substrate Gsk3β, but not Tsc2 [70], suggesting that Akt activity on Appl1-positive early endosomes promotes local Gsk3β phosphorylation. This hypothesis was recently supported by studies in non-neuronal cells, where Gsk3β phosphorylation by Akt was shown to activate clathrin-mediated endocytosis [76] and promote tumor cell migration and metastasis [77]. According to the model proposed by the authors [76,78], local Akt activation on early endosomes leads to Gsk3β phosphorylation, resulting in its inhibition and relief of an inhibitory phosphorylation of its substrate dynamin-1 (Figure 3). Notably, in agreement with the zebrafish study [70], interference with maturation of early endosomes resulted in strong inhibition of Akt-mediated phosphorylation of Gsk3β, but not Tsc2 [76], demonstrating that Akt partitioning to early endosomes restricts Akt substrate specificity to Gsk3β (Figure 2). Similarly, in *PI3K-C2γ^-/-^* mice, which display reduced production of PI(3,4)P_2_ on early endosomes, phosphorylation of GSK3β and its substrate, glycogen synthase, was markedly reduced, whereas phosphorylation of other Akt substrates, such as Tsc2 or FoxO1/FoxO3a, remained unaffected [65]. Conversely, phosphorylation of Tsc2 appears to be restricted to the late endosomes/lysosomes [79,80], although whether lysosomal localization of Akt is sufficient for Tsc2 phosphorylation is currently unknown. Finally, phosphorylation of hexokinase-2 by Akt was shown to stimulate its binding to mitochondria and protect insulin-responsive tissues from apoptotic stimuli (reviewed in [81], Figure 3). These examples demonstrate that partitioning of active Akt to cellular membranes could help restrict its substrate specificity.

Finally, compartmentalization of Akt substrates may also define the functional outcome of Akt activation at the subcellular compartments. Thus, a study using Akt pseudosubstrate inhibitors targeted to subcellular compartments suggested that nuclear and plasma membrane Akt pools specifically contribute to expression of the Glut4 transporter and differentiation of pre-adipocytes [82]. Another report showed that, while Akt phosphorylation on T308 was sufficient for inducing Glut4-mediated glucose uptake in fat and muscle cells in response to insulin, additional phosphorylation by mTORC2 was required for recruiting the ubiquitous Glut1 transporter in other cell types [83]. This findings suggests that intracellular mTORC2 pools [23] may contribute to defining the functional outcomes of Akt signaling. Further, local activation of Akt on lysosomal membranes was shown to inhibit the chaperone-mediated autophagy (CMA) by phosphorylating the lysosomal Akt substrate GFAP [84]. In this case, inactivation of Akt through recruitment of the phosphatase PHLPP1 and the corresponding decrease of phospho-GFAP resulted in CMA. Although the authors did not address whether CMA-inducing conditions correlated with a global decrease in phosphorylation of Akt substrates, it is tempting to speculate that different subcellular pools of Akt, each with a specific complement of the activating lipids, kinases, and inactivating phosphatases, could drive distinct functional outcomes.

## 5. Open Questions

Recent biochemical and cell-based studies, together with radically improved methods to monitor lipid dynamics with high temporal and spatial resolution, have offered a better framework for understanding Akt regulation and signaling in cells. Kinase-intrinsic allosteric activation of Akt by PI3K lipid products combined with its rapid inactivation by cellular phosphatases upon dissociation from the cellular membranes ensure tight coupling between Akt and the upstream PI3Ks. This coupling restricts Akt activity to PI(3,4,5)P_3_/PI(3,4)P_2_-containing cellular membrane compartments, determines the kinetics of Akt activation and quenching, and drives distinct functional responses by recruiting specific isoforms to a particular set of substrates. Dysregulation of the coupling, either through mutations at the Akt allosteric surface or overproduction of PI3K products, results in Akt hyperactivation and is often transforming.

Despite the progress in understanding the mechanisms of signaling fidelity through the PI3K/Akt axis, many important questions remain unanswered. Thus, it is not yet clear whether binding to PI3K products differentially affects the efficiency of Akt activation by upstream kinases. The interplay between Akt-specific and lipid phosphatases and their regulation by PI3K are currently not well understood. Furthermore, the ultimate test for the allosteric switch model will be the direct demonstration of the distinct kinetics of phosphorylation of Akt cytosolic and membrane-bound substrates. It is tempting to speculate that restriction of Akt activity to cellular membrane compartments may help define Akt substrate specificity and increase the processivity of substrate phosphorylation; detailed biochemical studies will be required to test this intriguing possibility. Finally, more studies will be needed to examine the alternative activation routes bypassing the PI3K-dependent allosteric switch, especially in conditions of pathologic Akt hyperactivation. Examining these questions from both biochemical and cell biological perspectives will further improve our understanding of how the organization of the PI3K/Akt axis in space and time determines fidelity of cellular signaling.
